# Minimally invasive glaucoma surgery (MIGS) devices: risks, benefits and suitability

**Published:** 2022-01-31

**Authors:** Francisco Otárola, Francisco Pooley

**Affiliations:** 1 Adjunct Professor of Ophthalmology: Specialty Department, Universidad de la Frontera School of Medicine, Temuco, Chile.; 2 Adjunct Professor of Ophthalmology: University of Chile, Hospital del Salvador, Santiago, Chile.

**Minimally invasive glaucoma surgery (MIGS) devices can be helpful in managing intraocular pressure in the early stages of glaucoma, thereby reducing patients’ reliance on medication. However, the IOP reduction tends to be small and the devices are expensive.**

Minimally invasive glaucoma surgery (MIGS) has emerged in the past few years as a relevant therapeutic option for glaucoma. Intraocular pressure (IOP) reduction is still the only proven treatment to halt glaucoma progression.^1^ This has been traditionally achieved by both nonsurgical means (topical medications or laser therapy) and surgical means (trabeculectomy or glaucoma drainage devices). None of these methods are ideal: compliance is the main issue for medications and surgical complications are common. The high safety profile of MIGS allows it to be used earlier than conventional types of glaucoma surgery within a glaucoma treatment plan, and is typically combined with cataract surgery in patients with mild to moderate primary open-angle glaucoma (POAG).^2^

MIGS usually involves the use of a small device that is inserted or placed through a clear corneal incision approached from inside the eye (ab interno). This allows for minimal tissue disruption, a more favorable risk profile, and faster recovery compared to conventional trabeculectomy or glaucoma drainage device implantation (Figure 1).

**Figure 1:**
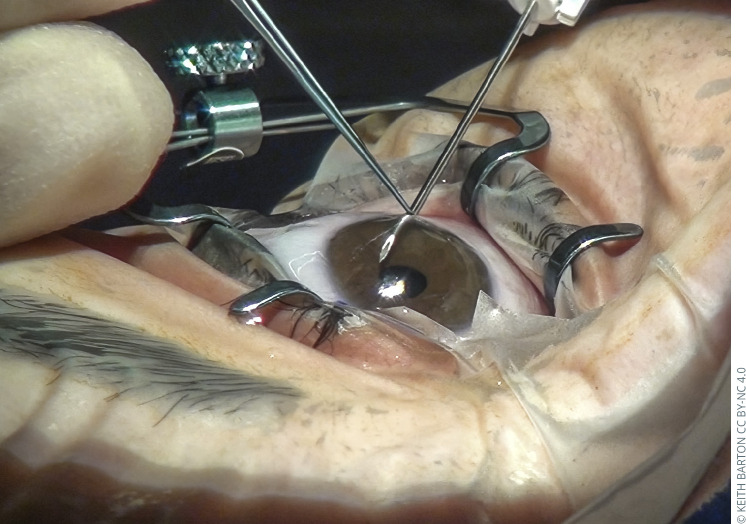
A minimally invasive glaucoma surgery device is implanted through the anterior chamber.

The benefit is that MIGS tends to be relatively safe and low risk. However, the IOP reduction tends to be small and there is no good evidence for their utility in low- and/or middle-income countries, where patients might be diagnosed with glaucoma at a very advanced stage.

Currently, there are many choices for the glaucoma surgeon where MIGS devices are concerned. They can be divided according to their site of action or placement: Schlemm’s canal, suprachoroidal, and subconjunctival.

**1. Schlemm’s canal devices**

*Trabectome, ELT (excimer laser trabeculotomy), iStent, iStent inject, Hydrus, and KDB (Kahook dual blade)*

Schlemm’s canal devices are inserted through an ab interno method with the assistance of a gonioscopic lens, aiming to increase aqueous humor outflow through the conventional pathway. Therefore, the potential effect on aqueous ouflow is influenced by the resistance provided by the episcleral venous pressure (Figure 2).

**Figure 2:**
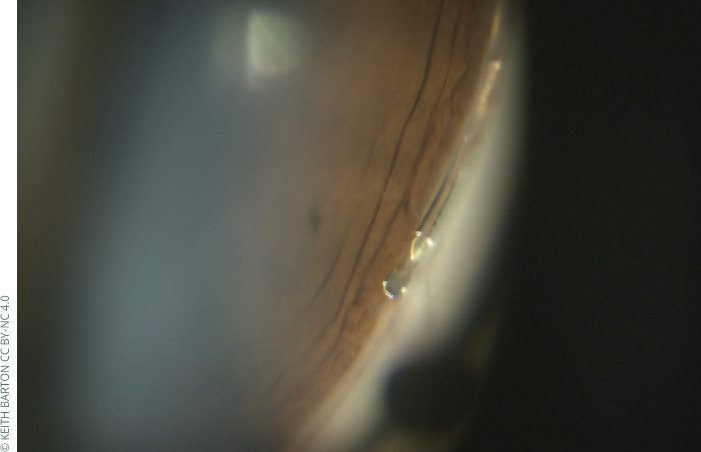
A Hydrus device implanted into Schlemm’s canal.

The most common procedures include the removal of trabecular tissue (Trabectome, ELT, KDB) or the implantation of a small device (iStent, iStent inject, Hydrus).

Among the products currently available, randomised clinical trial data associated the Hydrus with greater eye drop-free glaucoma control and IOP lowering than the iStent; however, these effect sizes were small.^3^^,^^4^

**2. Suprachoroidal devices**

*CyPass and iStent Supra*

Unlike the Schlemm’s canal devices, in which aqueous outflow could be affected by episcleral venous pressure, the suprachoroidal space is a potential space that confers minimal resistance to aqueous outflow. It allows aqueous to traverse the sclera directly via the intercellular spaces between ciliary muscle febres and loose connective tissue of the suprachoroidal space.

At present, there are no suprachoroidal devices clinically available, given that the CyPass MicroStent, despite receiving FDA approval in 2016, was withdrawn from the market after results from a post-marketing study showing accelerated endothelial cells loss.^5^ The iStent Supra is still undergoing investigation.

**3. Subconjuntival devices**

*XEN-45 and PreserFlo Microshunt*

The subconjunctival space, despite not being part of the physiological outflow pathway, is the drainage pathway most familiar to glaucoma surgeons as it is used in conventional glaucoma surgery. Just like the suprachoroidal space, the subconjunctival space is a potential site which is not limited by the episcleral venous pressure; however, aqueous drainage can be compromised by fibrosis and scarring.^6^

The XEN-45 gel stent is a biocompatible, hydrophilic tube made from porcine gelatin cross-linked with glutaraldehyde. It has been implanted using various techniques (ab-externo/ab-interno, with or without conjunctival peritomy).

The PreserFlo Microshunt is implanted through and ab-externo approach requiring conjunctival dissection. Despite this fact, is has been classified by the FDA as a MIGS device (Figure 3).

**Figure 3:**
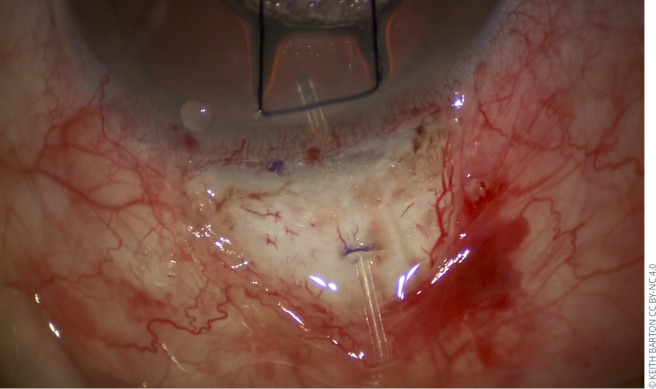
Testing the PreserFlo MIGS device before closing the conjunctiva.

Both devices are ‘bleb-forming’: designed to limit or prevent clinically significant postoperative hypotony. On the other hand, this may lead to significant scarring and device failure, the risk of which can be minimised by using antimetabolites and aggressive topical anti-inflammatory therapy in the postoperative period.

## Discussion

The overall modest reduction in IOP and generally favorable safety profile of Schlemm’s canal devices make it a welcome option for patients with mild or moderate glaucoma who would like to reduce their medication burden. Suprachoroidal and subconjunctival devices offer the potential of greater IOP reduction. There are no commercially available suprachoroidal devices and they are also potentially associated with unpredictable IOP spikes and hypotony. Subconjunctival devices may fail as a consequence of subconjunctival fibrosis or result in bleb-related complications.

There are a few key points to bear in mind when considering use of MIGS devices in areas of the world with limited resources for health care. Patients may present with very advanced glaucoma, and MIGS devices are likely to be less effective in these group of patients. Also, trials to date have been limited to patients with early to moderate disease.

**“There are a few key points to bear in mind when considering use of MIGS devices in areas of the world with limited resources for health care.”**

Conventional glaucoma surgery is still the gold standard for surgical management of glaucoma, and no MIGS device has been compared head-to-head with trabeculectomy or aqueous shunt in a randomised controlled trial.

Finally, MIGS devices are relatively expensive and therefore less likely to be a practical option in countries with limited resources. Some glaucoma drainage devices cost as little as US $50, compared to US $400 or more for any MIGS device; this also doesn’t take into account the extra cost of surgical goniolenses or the steep learning curve/training required for this type of surgery.

More prospective randomised trials, with longer follow-up periods, are required to further evaluate the efficacy and safety of this rapidly evolving field of glaucoma treatment. Further comparative studies between devices would also be helpful to evaluate their relative efficacy.
